# Associations Between Gestational Weight Gain and Adverse Birth Outcomes: A Population-Based Retrospective Cohort Study of 9 Million Mother-Infant Pairs

**DOI:** 10.3389/fnut.2022.811217

**Published:** 2022-02-14

**Authors:** Xue Liu, Huan Wang, Liu Yang, Min Zhao, Costan G. Magnussen, Bo Xi

**Affiliations:** ^1^Department of Epidemiology, School of Public Health, Qilu Hospital, Cheeloo College of Medicine, Shandong University, Jinan, China; ^2^Department of Nutrition and Food Hygiene, School of Public Health, Cheeloo College of Medicine, Shandong University, Jinan, China; ^3^Menzies Institute for Medical Research, University of Tasmania, Hobart, TAS, Australia; ^4^Research Centre of Applied and Preventive Cardiovascular Medicine, University of Turku, Turku, Finland; ^5^Centre for Population Health Research, University of Turku and Turku University Hospital, Turku, Finland

**Keywords:** gestational weight gain, adverse birth outcomes, pre-pregnancy obesity, pregnancy, birth cohort

## Abstract

**Background:**

Gestational weight gain (GWG) reflects maternal nutrition during pregnancy. However, the associations between maternal GWG and adverse birth outcomes are inconclusive.

**Objective:**

We aimed to examine the associations between maternal GWG and adverse birth outcomes according to maternal pre-pregnancy body mass index (BMI) categories in a large, multiethnic and diverse population in the U.S.

**Study Design:**

We used nationwide birth certificate data from the National Vital Statistics System to examine the association of GWG (below, within and above the Institute of Medicine [IOM] guidelines) with six adverse birth outcomes (preterm birth, low birthweight, macrosomia, small for gestational age [SGA], large for gestational age [LGA], and low Apgar score) according to the pre-pregnancy BMI categories (underweight to obesity grade 3). Multivariable logistic regression analyses were performed to estimate the odds ratios (ORs) and 95% confidence intervals (CIs).

**Results:**

A total of 9,191,842 women aged 18–49 years at delivery with live singleton births were included. Among them, 24.5% of women had GWG below IOM guidelines, 27.6% within the guidelines, and 47.9% above the guidelines. Compared with maternal GWG within guidelines, GWG below guidelines was associated with higher odds of preterm birth (OR = 1.52, 95%CI = 1.51–1.53), low birthweight (OR = 1.46, 95%CI = 1.45–1.47) and SGA (OR = 1.44, 95%CI = 1.43–1.45). In contrast, maternal GWG above guidelines was associated with higher odds of macrosomia (OR = 2.12, 95%CI = 2.11–2.14) and LGA (OR = 2.12, 95%CI = 2.11–2.14). In addition, maternal GWG below or above guidelines had slightly higher odds of low Apgar score (below guidelines: OR = 1.04, 95%CI = 1.03–1.06, above guidelines: OR = 1.17, 95%CI = 1.15–1.18). The results were largely similar among women with GWG below or above guidelines across pre-pregnancy BMI categories of underweight, overweight, and obesity grade 1 to grade 3.

**Conclusion:**

Pregnant women with GWG below or above the IOM guidelines have increased odds of selected adverse infant birth outcomes. Monitoring maternal GWG could enable physicians to provide tailored nutrition and exercise advice as well as prenatal care to pregnant women to reduce the likelihood of adverse birth outcomes.

## Introduction

Maternal pre-pregnancy body mass index (BMI) and gestational weight gain (GWG) reflect the maternal nutrition before and during pregnancy, which are considered important predictors of adverse perinatal outcomes for mothers and infants (1). Accumulating evidence suggests that higher maternal pre-pregnancy BMI is associated with increased risk of eclampsia, gestational hypertension and diabetes for mothers, and large-for-gestational-age (LGA) and macrosomia for infants (2). In addition, lower pre-pregnancy BMI is associated with increased odds of preterm birth, low birthweight and small-for-gestational-age (SGA) (3). Moreover, there are data that suggest insufficient maternal GWG is associated with preterm birth, low birthweight and SGA whereas excessive maternal GWG might be associated with macrosomia and LGA—although the findings have been inconsistent (2, 4–7). While meta-analyses have been used in an attempt to address the inconsistent findings, there was significant between-study heterogeneity to draw convincing conclusions (4, 5). Moreover, maternal GWG may vary by maternal age and race/ethnicity (8, 9). However, to our knowledge, few studies have assessed the association between maternal GWG and adverse birth outcomes by maternal age and race/ethnicity.

In this study, we aimed to examine the associations of maternal GWG (below, within and above the 2009 Institute of Medicine [IOM] guidelines) with adverse birth outcomes (preterm birth, low birthweight, macrosomia, SGA, LGA, and low Apgar score) according to maternal pre-pregnant BMI categories in a large, multiethnic and population-based cohort.

## Materials and Methods

### Study Population

This study used nationwide birth certificate data from the National Vital Statistics System (NVSS) (2016–2018), which is a U.S. population-based retrospective cohort study from 50 States and the District of Columbia. The NVSS is a major cooperative effort between the U.S. Centers for Disease Control and Prevention (CDC) and all U.S. states, which gathers information on maternal exposures before and during pregnancy and infant outcomes at delivery using two uniform documents: a facility worksheet and a maternal worksheet. Detailed methods, quality control, and vital statistics can be found on the CDC website (https://www.cdc.gov/nchs/nvss/births.htm). The de-identified data are publically available online, so the ethical board review of the corresponding author's institution is exempted.

**Figure 1:**
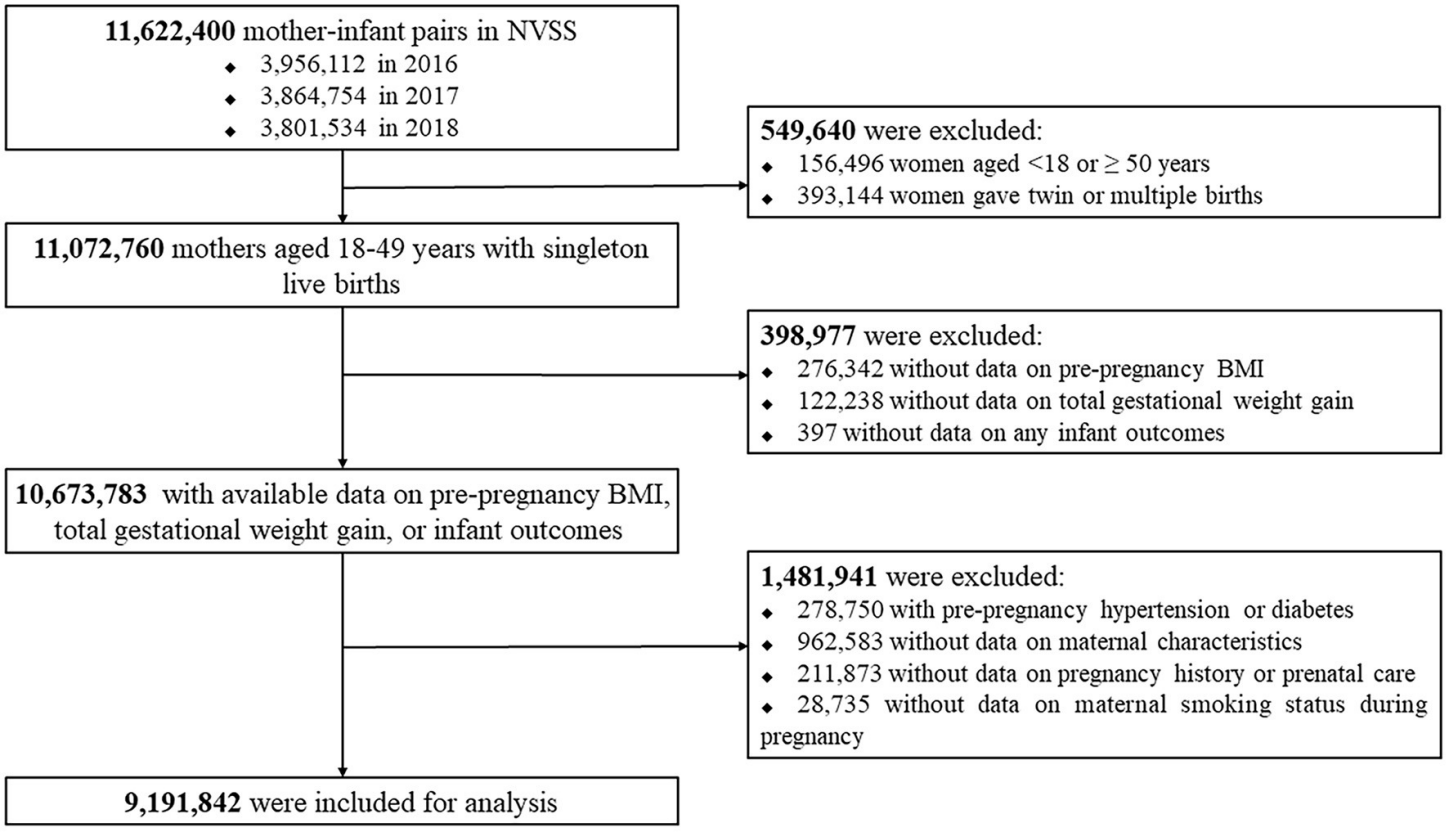
Flow chart of the population selection process.

We firstly included all mother-infant pairs (*n* = 11,622,400) in the NVSS between 2016 and 2018 because all U.S. States and the District of Columbia had fully implemented the 2003 version of Standard Certificate of Live Birth to collect the birth information since 2016. We then excluded women aged <18 or ≥ 50 years (*n* = 156,496) at delivery or who delivered twin/multiple births (*n* = 393,144), women with missing data on pre-pregnancy BMI (*n* = 276,342), total GWG (*n* = 122,238) or any infant outcomes (*n* = 397), women with pre-pregnancy hypertension or diabetes (*n* = 278,750), and women with missing data on maternal characteristics (*n* = 962,583), pregnancy history or prenatal care (*n* = 211,873), or maternal smoking status during pregnancy (*n* = 28,735). Following these exclusions, 9,191,842 women with live singleton births were included in the analysis. Figure 1 presents the flow chart of the inclusion/exclusion of the participants. The study followed the reporting guidelines in the Statement of Strengthening the Reporting of Observational Studies in Epidemiology (STROBE) (10).

### Maternal GWG and Pre-pregnancy BMI

Maternal GWG was calculated as weight at delivery (from medical records) minus pre-pregnancy weight (i.e., weight immediately before the mother became pregnant with this child), and categorized as below, within, or above the 2009 IOM guidelines (11). We classified maternal pre-pregnancy BMI as underweight (BMI <18.5 kg/m^2^), normal weight (18.5–24.9 kg/m^2^), overweight (25–29.9 kg/m^2^) or, obesity (≥30 kg/m^2^) according to the U.S. National Heart, Lung, and Blood Institutes categories (12). Obesity was further subdivided into obesity grade 1 (30–34.9kg/m^2^), obesity grade 2 (35–39.9kg/m^2^), and obesity grade 3 (≥40kg/m^2^) (13).

### Adverse Infant Birth Outcomes

We analyzed six adverse infant birth outcomes, including preterm birth, low birthweight, macrosomia, SGA, LGA, and low Apgar score. Preterm birth was defined as <37 weeks of gestational age at birth. Low birthweight was defined as birthweight <2,500 g and macrosomia as birthweight >4,000 g. SGA and LGA at birth were defined as sex- and gestational age-specific birthweight <10th percentile and >90th percentile, respectively, according to the U.S. new intrauterine growth curves (14). The Apgar score was usually evaluated at 1, 5, and 10 min after birth, indicating the neonatal overall status and response to resuscitation at specific intervals (15). A 5-min Apgar score is predictive for neonatal survival and has a range of 0 to 10 (16). Low Apgar score was identified as a 5-min Apgar score < 7, indicating a low physical condition.

### Study Covariates

Study covariates included maternal age at delivery, race/ethnicity, education level, marital status, smoking status during pregnancy, parity, infant sex, and total number of prenatal care visits. Maternal age at delivery was categorized as 18–29, 30–39, and 40–49 years old. Maternal race/ethnicity was divided into Hispanic, non-Hispanic white, non-Hispanic black, and other. Maternal highest education level was divided into less than high school, high school, and more than high school. Marital status was reported as “married” or “unmarried.” Smoking status during pregnancy was recorded as “yes” or “no.” Parity indicated the number of live births that a mother had, including this delivery. Infant sex was defined as male or female, and total number of prenatal care visits was categorized as 0, 1–4, 5–9, and ≥10. Gestational age at delivery was calculated based on the obstetric estimate of gestation.

### Statistical Analyses

Baseline characteristics of the study population were presented according to maternal pre-pregnancy BMI category. We used median (interquartile range, IQR) for continuous variables (normality test *P* < 0.05 for all continuous variables) and *n* (%) for categorical variables. The differences of sociodemographic characteristics between pre-pregnancy BMI groups were assessed using Kruskal-Wallis test or Chi-square test. Multivariable logistic regression models were performed to estimate the odds ratios (ORs) with 95% confidence intervals (CIs) for the associations of maternal GWG below or above guidelines (using values within guidelines as the reference group) with adverse infant birth outcomes with adjustment for maternal age at delivery, race/ethnicity, education level, marital status, smoking status during pregnancy, parity, infant sex, and total number of prenatal care visits. Evidence for a dose-response relationship between maternal GWG (as a continuous variable) and infant birth outcomes were assessed by restricted cubic spline logistic regression models with adjustment for all potential covariates, with three knots at the 5th, 50th, and 95th percentiles. To assess the modifying effects of race/ethnicity, maternal age at delivery and infant birth year on the associations, subgroup analyses were performed that stratified by these three variables. To assess the robustness of our findings, two sensitivity analyses were performed, including the exclusion of women with cesarean section, and the exclusion of women with eclampsia, gestational hypertension or diabetes. All analyses were performed in SAS 9.4. A two-sided *P*-value of < 0.05 was considered statistically significant.

## Results

### Characteristics of the Study Population

Table 1 shows the baseline characteristics of the study population by maternal pre-pregnancy BMI category. At baseline, among all 9,191,842 pregnant women, 309,198 (3.4%) were categorized as underweight, 4,037,594 (43.9%) as normal weight, 2,432,107 (26.5%) as overweight, 1,338,053 (14.6%) were obesity grade 1, 642,534 (7.0%) as obesity grade 2, and 432,356 (4.7%) as obesity grade 3. Overall, 2,250,436 (24.5%) of the study population had total GWG below IOM guidelines, 2,537,994 (27.6%) within guidelines, and 4,403,412 (47.9%) above guidelines. Compared with women of pre-pregnancy normal weight, those with pre-pregnancy overweight and obesity were more likely to have GWG above guidelines, whereas those with underweight were less likely to have GWG above guidelines; the patterns were opposite for women with GWG below guidelines. Overall, the prevalence estimates of preterm birth, low birthweight, macrosomia, SGA, LGA, and low Apgar score were 7.5, 6.1, 8.1, 5.7, 7.0, and 1.8%, respectively.

**Table 1:** Characteristics of the study population by maternal pre-pregnancy BMI category.

**Characteristics**	**Total**	**Underweight**	**Normal weight**	**Overweight**	**Obesity**	**Obesity grade 1**	**Obesity grade 2**	**Obesity grade 3**	*P*-value^*^
*N*	9191842	309198	4037594	2432107	2412943	1338053	642534	432356	
Pre-pregnancy BMI, kg/m^2^, median (IQR)	25.4 (22.1–30.2)	17.7 (17.0–18.1)	22.1 (20.7–23.5)	27.1 (25.8–28.3)	34.3 (31.8–38.3)	32.0 (30.9–33.3)	37.0 (35.9–38.3)	43.4 (41.5–46.6)	<0.001
Total GWG, kg, median (IQR)	13.2 (9.1–17.2)	14.1 (11.3–18.1)	14.1 (10.9–18.1)	13.2 (9.1–17.7)	10.0 (5.4–15.0)	11.3 (6.8–15.9)	9.1 (4.5–14.1)	7.7 (3.2–12.7)	<0.001
GWG status, *n* (%)									<0.001
Below guidelines	2250436 (24.5)	107610 (34.8)	1184681 (29.3)	387406 (15.9)	570739 (23.7)	238465 (17.8)	173829 (27.1)	158445 (36.7)	
Within guidelines	2537994 (27.6)	120947 (39.1)	1385700 (34.3)	593060 (24.4)	438287 (18.2)	233007 (17.4)	123008 (19.1)	82272 (19.0)	
Above guidelines	4403412 (47.9)	80641 (26.1)	1467213 (36.3)	1451641 (59.7)	1403917 (58.2)	866581 (64.8)	345697 (53.8)	191639 (44.3)	
Maternal age at delivery, years									
Median (IQR)	29 (25–33)	26 (22–31)	29 (25–33)	29 (25–33)	29 (25–33)	29 (25–33)	28 (25–33)	28 (25–33)	<0.001
Category, *n* (%)									<0.001
18–29	5050128 (54.9)	211121 (68.3)	2183261 (54.1)	1301405 (53.5)	1354341 (56.1)	742295 (55.5)	365322 (56.9)	246724 (57.1)	
30–39	3877950 (42.2)	93248 (30.2)	1745708 (43.2)	1052741 (43.3)	986253 (40.9)	553398 (41.4)	258513 (40.2)	174342 (40.3)	
40-49	263764 (2.9)	4829 (1.6)	108625 (2.7)	77961 (3.2)	72349 (3.0)	42360 (3.2)	18699 (2.9)	11290 (2.6)	
Race/ethnicity, *n* (%)									<0.001
Hispanic	1984529 (21.6)	50462 (16.3)	757061 (18.8)	603098 (24.8)	573908 (23.8)	341892 (25.6)	147010 (22.9)	85006 (19.7)	
Non-Hispanic white	5063936 (55.1)	167284 (54.1)	2396688 (59.4)	1271631 (52.3)	1228333 (50.9)	670594 (50.1)	333438 (51.9)	224301 (51.9)	
Non-Hispanic black	1322216 (14.4)	41853 (13.5)	446526 (11.1)	362021 (14.9)	471816 (19.6)	240710 (18.0)	128222 (20.0)	102884 (23.8)	
Other	821161 (8.9)	49599 (16.0)	437319 (10.8)	195357 (8.0)	138886 (5.8)	84857 (6.3)	33864 (5.3)	20165 (4.7)	
Education level, *n* (%)									<0.001
Less than high school	1112762 (12.1)	47134 (15.2)	434945 (10.8)	317239 (13.0)	313444 (13.0)	181986 (13.6)	80620 (12.6)	50838 (11.8)	
High school	2359608 (25.7)	95799 (31.0)	925568 (22.9)	619270 (25.5)	718971 (29.8)	383652 (28.7)	195766 (30.5)	139553 (32.3)	
More than high school	5719472 (62.2)	166265 (53.8)	2677081 (66.3)	1495598 (61.5)	1380528 (57.2)	772415 (57.7)	366148 (57.0)	241965 (56.0)	
Marital status, *n* (%)									<0.001
Married	5623379 (61.2)	164902 (53.3)	2634517 (65.3)	1486058 (61.1)	1337902 (55.5)	759219 (56.7)	352507 (54.9)	226176 (52.3)	
Unmarried	3568463 (38.8)	144296 (46.7)	1403077 (34.8)	946049 (38.9)	1075041 (44.6)	578834 (43.3)	290027 (45.1)	206180 (47.7)	
Smoking during pregnancy, *n* (%)									<0.001
Yes	670778 (7.3)	39469 (12.8)	274469 (6.8)	162276 (6.7)	194564 (8.1)	102426 (7.7)	53984 (8.4)	38154 (8.8)	
No	8521064 (92.7)	269729 (87.2)	3763125 (93.2)	2269831 (93.3)	2218379 (91.9)	1235627 (92.4)	588550 (91.6)	394202 (91.2)	
Parity, *n* (%)									<0.001
1	3481568 (37.9)	142754 (46.2)	1676131 (41.5)	871382 (35.8)	791301 (32.8)	439672 (32.9)	209811 (32.7)	141818 (32.8)	
2	2991287 (32.5)	95671 (30.9)	1317924 (32.6)	794585 (32.7)	783107 (32.5)	433430 (32.4)	208844 (32.5)	140833 (32.6)	
3	1581876 (17.2)	43378 (14.0)	635697 (15.7)	438887 (18.1)	463914 (19.2)	257218 (19.2)	123400 (19.2)	83296 (19.3)	
≥4	1137111 (12.4)	27395 (8.9)	407842 (10.1)	327253 (13.5)	374621 (15.5)	207733 (15.5)	100479 (15.6)	66409 (15.4)	
Infant sex, *n* (%)									0.072
Male	4702750 (51.2)	157598 (51.0)	2065638 (51.2)	1245693 (51.2)	1233821 (51.1)	684681 (51.2)	328203 (51.1)	220937 (51.1)	
Female	4489092 (48.8)	151600 (49.0)	1971956 (48.8)	1186414 (48.8)	1179122 (48.9)	653372 (48.8)	314331 (48.9)	211419 (48.9)	
Total number of prenatal care visits, *n* (%)									<0.001
0	137691 (1.5)	6761 (2.2)	62224 (1.5)	36426 (1.5)	32280 (1.3)	18951 (1.4)	8200 (1.3)	5129 (1.2)	
1–4	336482 (3.7)	15010 (4.9)	144502 (3.6)	89434 (3.7)	87536 (3.6)	49657 (3.7)	22768 (3.5)	15111 (3.5)	
5–9	1907653 (20.8)	72627 (23.5)	834855 (20.7)	507277 (20.9)	492894 (20.4)	278683 (20.8)	128929 (20.1)	85282 (19.7)	
≥10	6810016 (74.1)	214800 (69.5)	2996013 (74.2)	1798970 (74.0)	1800233 (74.6)	990762 (74.1)	482637 (75.1)	326834 (75.6)	
Preterm birth	686732 (7.5)	29376 (9.5)	275860 (6.8)	175087 (7.2)	206409 (8.6)	108062 (8.1)	56204 (8.8)	42143 (9.8)	<0.001
Birthweight category									<0.001
Low (<2500 g)	557335 (6.1)	33955 (11.0)	244399 (6.1)	134720 (5.5)	144261 (6.0)	78196 (5.9)	38599 (6.0)	27466 (6.4)	
Normal (2500-4000 g)	7886957 (85.9)	266519 (86.3)	3536000 (87.6)	2081787 (85.7)	2002651 (83.1)	1123828 (84.1)	529783 (82.5)	349040 (80.8)	
Macrosomia (>4000 g)	741473 (8.1)	8484 (2.8)	254513 (6.3)	214141 (8.8)	264335 (11.0)	135133 (10.1)	73698 (11.5)	55504 (12.9)	
Birthweight status for sex and gestational age									<0.001
Small for gestational age	521691 (5.7)	34105 (11.1)	252137 (6.3)	122789 (5.1)	112660 (4.7)	64322 (4.8)	29636 (4.6)	18702 (4.4)	
Appropriate for gestational age	7983949 (87.3)	266676 (86.6)	3567324 (88.8)	2115988 (87.4)	2033961 (84.7)	1143066 (85.8)	537711 (84.1)	353184 (82.1)	
Large for gestational age	641816 (7.0)	7094 (2.3)	198140 (4.9)	181844 (7.5)	254738 (10.6)	124288 (9.3)	72091 (11.3)	58359 (13.6)	
Low Apgar score (<7)	160577 (1.8)	5062 (1.6)	61393 (1.5)	41535 (1.7)	52587 (2.2)	26076 (2.0)	14554 (2.3)	11957 (2.8)	<0.001

*BMI, body mass index; IQR, interquartile range; GWG, gestational weight gain*.

* *Differences in characteristics across 6 pre-pregnancy BMI categories were assessed using Kruskal-Wallis test for continuous variables, and Chi-square test for categories variable*.

### Associations of GWG With Infant Birth Outcomes

#### Preterm Birth

Compared with women with GWG within guidelines, those with GWG below guidelines had increased odds for preterm birth, with highest odds among women with pre-pregnancy underweight. The ORs (95% CIs) of preterm birth were 2.12 (2.06–2.18) for underweight, 1.76 (1.74–1.77) for normal weight, 1.33 (1.31–1.35) for overweight, 1.15 (1.12–1.17) for obesity grade 1, 1.17 (1.14–1.20) for obesity grade 2, and 1.12 (1.09–1.15) for obesity grade, 3. However, those above guidelines had decreased odds for preterm birth with lowest odds among women with pre-pregnancy underweight. The ORs (95% CIs) of preterm birth were 0.68 (0.66–0.71) for underweight, 0.72 (0.72–0.73) for normal weight, 0.70 (0.70–0.71) for overweight, 0.78 (0.77–0.79) for obesity grade 1, 0.88 (0.86–0.90) for obesity grade 2, and 0.96 (0.93–0.99) for obesity grade 3 (Tables 2, 3).

**Table 2:** Association of gestational weight gain below or above guidelines with Infant birth outcomes by maternal pre-pregnancy BMI category.

**Birth outcomes**	**Total**		**Underweight**		**Normal weight**		**Overweight**		**Obesity**	
	***n* (%)**	**OR (95% CI)**	***n* (%)**	**OR (95% CI)**	***n* (%)**	**OR (95% CI)**	***n* (%)**	**OR (95% CI)**	***n* (%)**	**OR (95% CI)**
**Preterm birth**										
Below guidelines	255140 (11.3)	1.52 (1.51–1.53)	16444 (15.3)	2.12 (2.06–2.18)	132022 (11.2)	1.76 (1.74–1.77)	45722 (11.8)	1.33 (1.31–1.35)	60952 (10.7)	1.15 (1.14–1.17)
Within guidelines	177442 (7.0)	1.00	8609 (7.1)	1.00	80437 (5.8)	1.00	48479 (8.2)	1.00	39917 (9.1)	1.00
Above guidelines	254150 (5.8)	0.83 (0.82–0.83)	4323 (5.4)	0.68 (0.66-0.71)	63401 (4.3)	0.72 (0.72–0.73)	80886 (5.6)	0.70 (0.70–0.71)	105540 (7.5)	0.83 (0.82–0.84)
**Low birthweight**										
Below guidelines	240501 (10.7)	1.46 (1.45–1.47)	20357 (18.9)	1.88 (1.82–1.94)	130683 (11.0)	1.59 (1.57–1.61)	41261 (10.7)	1.33 (1.30–1.36)	48200 (8.5)	1.18 (1.15–1.20)
Within guidelines	144450 (5.7)	1.00	9566 (7.9)	1.00	67467 (4.9)	1.00	38504 (6.5)	1.00	28913 (6.6)	1.00
Above guidelines	172384 (3.9)	0.69 (0.68–0.70)	4032 (5.0)	0.63 (0.60–0.66)	46249 (3.2)	0.69 (0.68-0.70)	54955 (3.8)	0.71 (0.70–0.72)	67148 (4.8)	0.78 (0.77–0.80)
**Macrosomia**										
Below guidelines	85604 (3.8)	0.75 (0.74–0.76)	962 (0.9)	0.48 (0.44–0.51)	31533 (2.7)	0.61 (0.60–0.62)	14244 (3.7)	0.72 (0.70–0.73)	38865 (6.8)	0.81 (0.80–0.82)
Within guidelines	148499 (5.9)	1.00	2899 (2.4)	1.00	72930 (5.3)	1.00	34583 (5.8)	1.00	38087 (8.7)	1.00
Above guidelines	507370 (11.5)	2.12 (2.11–2.14)	4623 (5.7)	2.52 (2.40–2.65)	150050 (10.2)	2.06 (2.04–2.08)	165314 (11.4)	1.93 (1.91–1.96)	187383 (13.4)	1.61 (1.59–1.63)
**Small for** **gestational age**										
Below guidelines	196684 (8.8)	1.44 (1.43–1.45)	17152 (16.0)	1.77 (1.73–1.82)	112738 (9.6)	1.57 (1.56–1.59)	31719 (8.3)	1.32 (1.30–1.34)	35075 (6.2)	1.18 (1.16–1.20)
Within guidelines	149155 (5.9)	1.00	11655 (9.7)	1.00	80403 (5.8)	1.00	34801 (5.9)	1.00	22296 (5.1)	1.00
Above guidelines	175852 (4.0)	0.66 (0.66–0.67)	5298 (6.6)	0.61 (0.59–0.63)	58996 (4.0)	0.65 (0.64–0.66)	56269 (3.9)	0.66 (0.65–0.67)	55289 (4.0)	0.76 (0.75–0.77)
**Large for** **gestational age**										
Below guidelines	86810 (3.9)	0.82 (0.81–0.83)	1354 (1.3)	0.70 (0.65–0.75)	30519 (2.6)	0.69 (0.68-0.70)	14619 (3.8)	0.77 (0.76–0.79)	40318 (7.1)	0.83 (0.82–0.84)
Within guidelines	125777 (5.0)	1.00	2292 (1.9)	1.00	55172 (4.0)	1.00	30716 (5.2)	1.00	37597 (8.6)	1.00
Above guidelines	429229 (9.8)	2.12 (2.11–2.14)	3448 (4.3)	2.40 (2.27–2.53)	112449 (7.7)	2.08 (2.06–2.10)	136509 (9.4)	1.89 (1.86–1.91)	176823 (12.6)	1.57 (1.55–1.59)
**Low Apgar score**										
Below guidelines	51086 (2.3)	1.04 (1.03–1.06)	2367 (2.2)	0.98 (0.92–1.06)	23664 (2.0)	1.01 (0.99–1.03)	9800 (2.5)	1.07 (1.04–1.10)	15255 (2.7)	1.05 (1.02–1.08)
Within guidelines	37888 (1.5)	1.00	1513 (1.3)	1.00	17502 (1.3)	1.00	9689 (1.6)	1.00	9184 (2.1)	1.00
Above guidelines	71603 (1.6)	1.17 (1.15–1.18)	1182 (1.5)	1.22 (1.13–1.32)	20227 (1.4)	1.14 (1.12–1.16)	22046 (1.5)	1.08 (1.06–1.11)	28148 (2.0)	1.09 (1.06–1.11)

*BMI, body mass index; CI, confidence interval; OR, odds ratio*.

*Logistic regression models were adjusted for maternal age at delivery, race/ethnicity, education level, marital status, smoking during pregnancy, parity, infant sex, and total number of prenatal care visits*.

*Gestational age was additionally adjusted for low birthweight, macrosomia, and low Apgar score*.

**Table 3:** Association between gestational weight gain below or above guidelines with infant outcomes by maternal pre-pregnancy obesity severity.

**Birth outcomes**	**Obesity grade 1**		**Obesity grade 2**		**Obesity grade 3**		*P*-value^*^
	***n* (%)**	**OR (95% CI)**	***n* (%)**	**OR (95% CI)**	***n* (%)**	**OR (95% CI)**	
**Preterm birth**							
Below guidelines	26056 (10.9)	1.15 (1.12–1.17)	18184 (10.5)	1.17 (1.14–1.20)	16712 (10.6)	1.12 (1.09–1.15)	<0.001
Within guidelines	21216 (9.1)	1.00	10911 (8.9)	1.00	7790 (9.5)	1.00	<0.001
Above guidelines	60790 (7.0)	0.78 (0.77–0.79)	27109 (7.8)	0.88 (0.86–0.90)	17641 (9.2)	0.96 (0.93–0.99)	<0.001
**Low birthweight**							
Below guidelines	22047 (9.3)	1.25 (1.21–1.28)	14124 (8.1)	1.18 (1.13–1.23)	12029 (7.6)	1.20 (1.14–1.25)	<0.001
Within guidelines	16070 (6.9)	1.00	7755 (6.3)	1.00	5088 (6.2)	1.00	<0.001
Above guidelines	40079 (4.6)	0.75 (0.73–0.77)	16720 (4.8)	0.78 (0.75–0.81)	10349 (5.4)	0.85 (0.81–0.89)	<0.001
**Macrosomia**							
Below guidelines	12312 (5.2)	0.77 (0.75–0.79)	12206 (7.0)	0.75 (0.73-0.77)	14347 (9.1)	0.74 (0.72–0.76)	<0.001
Within guidelines	16510 (7.1)	1.00	11750 (9.6)	1.00	9827 (12.0)	1.00	<0.001
Above guidelines	106311 (12.3)	1.79 (1.76–1.82)	49742 (14.4)	1.63 (1.60–1.67)	31330 (16.4)	1.53 (1.49–1.57)	<0.001
**Small for gestational age**							
Below guidelines	16691 (7.1)	1.23 (1.20–1.26)	10316 (6.0)	1.19 (1.15–1.23)	8068 (5.1)	1.18 (1.13–1.22)	<0.001
Within guidelines	12735 (5.5)	1.00	6014 (4.9)	1.00	3547 (4.3)	1.00	<0.001
Above guidelines	34896 (4.0)	0.72 (0.71–0.74)	13306 (3.9)	0.77 (0.75–0.79)	7087 (3.7)	0.83 (0.80–0.87)	0.267
**Large for gestational age**							
Below guidelines	12345 (5.2)	0.78 (0.76–0.80)	12455 (7.2)	0.77 (0.75–0.79)	15518 (9.9)	0.76 (0.74–0.78)	<0.001
Within guidelines	15889 (6.9)	1.00	11469 (9.4)	1.00	10239 (12.5)	1.00	<0.001
Above guidelines	96054 (11.1)	1.73 (1.70–1.76)	48167 (14.0)	1.64 (1.60–1.67)	32602 (17.1)	1.52 (1.48–1.55)	<0.001
**Low Apgar score**							
Below guidelines	6177 (2.6)	1.08 (1.04–1.13)	4527 (2.6)	1.02 (0.97–1.08)	4551 (2.9)	0.95 (0.90–1.00)	<0.001
Within guidelines	4437 (1.9)	1.00	2576 (2.1)	1.00	2171 (2.6)	1.00	<0.001
Above guidelines	15462 (1.8)	1.10 (1.06–1.14)	7451 (2.2)	1.15 (1.09–1.20)	5235 (2.7)	1.09 (1.03–1.15)	<0.001

*BMI, body mass index; CI, confidence interval; OR, odds ratio*.

*Logistic regression models were adjusted for maternal age at delivery, race/ethnicity, education level, marital status, smoking during pregnancy, parity, infant sex, and total number of prenatal care visits*.

*Gestational age was additionally adjusted for three outcomes including low birthweight/macrosomia/low Apgar score*.

* *Differences in the proportion of adverse birth outcomes across the pre-pregnancy grade of obesity were assessed using Chi-square test*.

#### Low Birth Weight

Compared with women with GWG within guidelines, those with GWG below guidelines had increased odds for low birthweight, with highest odds among women with pre-pregnancy underweight. The ORs (95% CIs) for low birthweight were 1.88 (1.82–1.94) for underweight, 1.59 (1.57–1.61) for normal weight, 1.33 (1.30–1.36) for overweight, 1.25 (1.21–1.28) for obesity grade 1, 1.18 (1.13–1.23) for obesity grade 2, and 1.20 (1.14–1.25) for obesity grade 3. However, those with GWG above guidelines had decreased odds for low birthweight, with lowest odds among women with pre-pregnancy underweight. The ORs (95% CIs) for low birthweight were 0.63 (0.60–0.66) for underweight, 0.69 (0.68–0.70) for normal weight, 0.71 (0.70–0.72) for overweight, 0.75 (0.73–0.77) for obesity grade 1, 0.78 (0.75–0.81) for obesity grade 2, and 0.85 (0.81–0.89) for obesity grade 3 (Tables 2, 3).

#### Macrosomia

Compared with women with GWG within guidelines, those with GWG below guidelines had decreased odds for macrosomia, with lowest odds among women with pre-pregnancy underweight. The ORs (95% CIs) for macrosomia were 0.48 (0.44–0.51) for underweight, 0.61 (0.60–0.62) for normal weight, 0.72 (0.70–0.73) for overweight, 0.77 (0.75–0.79) for obesity grade 1, 0.75 (0.73–0.77) for obesity grade 2, and 0.74 (0.72–0.76) for obesity grade 3. However, those with GWG above guidelines had increased odds for macrosomia, with highest odds among women with pre-pregnancy underweight. The ORs (95% CIs) of macrosomia were 2.52 (2.40–2.65) for underweight, 2.06 (2.04–2.08) for normal weight, 1.93 (1.91–1.96) for overweight, 1.79 (1.76–1.82) for obesity grade 1, 1.63 (1.60–1.67) for obesity grade 2, and 1.53 (1.49–1.57) for obesity grade 3 (Tables 2, 3).

#### SGA

Compared with women with GWG within guidelines, those with GWG below guidelines had increased odds for SGA, with highest odds among women with pre-pregnancy underweight. The ORs (95% CIs) for SGA were 1.77 (1.73–1.82) for underweight, 1.57 (1.56–1.59) for normal weight, 1.32 (1.30–1.34) for overweight, 1.23 (1.20–1.26) for obesity grade 1, 1.19 (1.15–1.23) for obesity grade 2, and 1.18 (1.13–1.22) for obesity grade 3. However, those with GWG above guidelines had decreased odds for SGA, with lowest odds among women with pre-pregnancy underweight. The ORs (95% CIs) for SGA were 0.61 (0.59–0.63) for underweight, 0.65 (0.64–0.66) for normal weight, 0.66 (0.65–0.67) for overweight, 0.72 (0.71–0.74) for obesity grade 1, 0.77 (0.75–0.79) for obesity grade 2, and 0.83 (0.80–0.87) for obesity grade 3 (Tables 2, 3).

#### LGA

Compared with women with GWG within guidelines, those with GWG below guidelines had decreased odds for LGA, with lowest odds among women with pre-pregnancy normal weight. The ORs (95% CIs) of LGA were 0.70 (0.65–0.75) for underweight, 0.69 (0.68–0.70) for normal weight, 0.77 (0.76–0.79) for overweight, 0.78 (0.76–0.80) for obesity grade 1, 0.77 (0.75–0.79) for obesity grade 2 and 0.76 (0.74–0.78) for obesity grade 3. However, those with GWG above guidelines had increased odds for LGA, with highest odds among women with pre-pregnancy underweight. The ORs (95% CIs) of LGA were 2.40 (2.27–2.53) for underweight, 2.08 (2.06–2.10) for normal weight, 1.89 (1.86–1.91) for overweight, 1.73 (1.70–1.76) for obesity grade 1, 1.64 (1.60–1.67) for obesity grade 2, and 1.52 (1.48–1.55) for obesity grade 3 (Tables 2, 3).

#### Low Apgar Score

Compared with women with GWG within guidelines, those with GWG below guidelines had slightly increased odds for low Apgar score especially for those with pre-pregnancy overweight, and obesity grade 1. The ORs (95% CIs) for low Apgar score were 0.98 (0.92–1.06) for underweight, 1.01 (0.99–1.03) for normal weight, 1.07 (1.04–1.10) for overweight, 1.08 (1.04–1.13) for obesity grade 1, 1.02 (0.97–1.08) for obesity grade 2, and 0.95 (0.90–1.00) for obesity grade 3. Similarly, those with GWG above guidelines had increased odds for low Apgar score, with highest odds among women with pre-pregnancy underweight. The ORs (95% CIs) for low Apgar score were 1.22 (1.13–1.32) for underweight, 1.14 (1.12–1.16) for normal weight, 1.08 (1.06–1.11) for overweight, 1.10 (1.06–1.14) for obesity grade 1, 1.15 (1.09–1.20) for obesity grade 2, and 1.09 (1.03–1.15) for obesity grade 3 (Tables 2, 3).

#### Dose-Response Relationships of Maternal GWG With Infant Birth Outcomes

**Figure 2:**
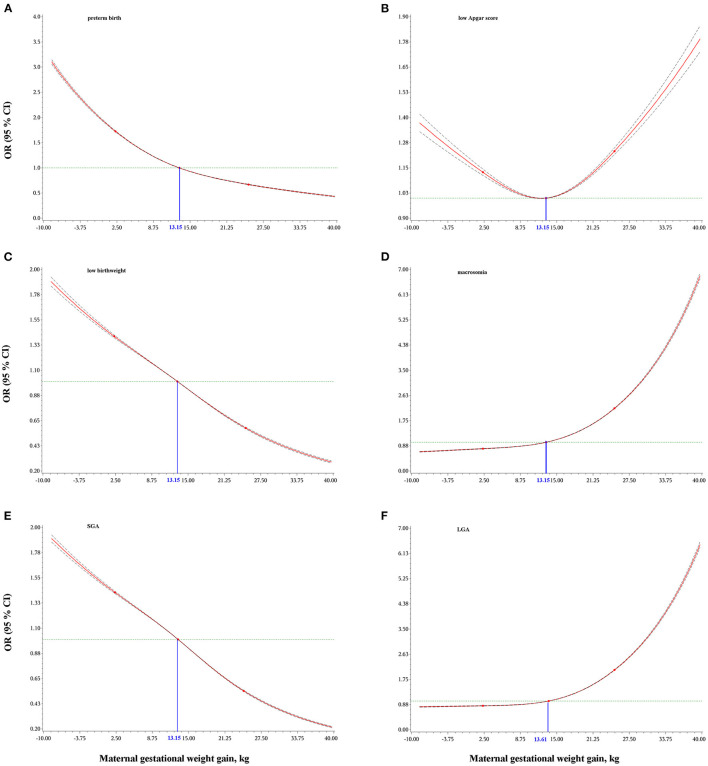
Dose-response relationships of maternal gestational weight gain with preterm birth **(A)**, low Apgar score **(B)**, low birthweight **(C)**, macrosomia **(D)**, small for gestational age, SGA **(E)**, and large for gestational age, LGA **(F)**. Odds ratios (OR) and 95% confidence intervals (CI) were calculated using logistic regression models with adjustment for maternal age at delivery, race/ethnicity, education level, marital status, smoking during pregnancy, parity, infant sex, and total number of prenatal care visits. Gestational age was additionally adjusted for low birthweight, macrosomia, and low Apgar score. The blue numbers indicate the median values of maternal gestational weight gain.

As shown in Figure 2, a lower GWG was associated with increased odds of preterm birth, low birthweight and SGA, while a higher gestational weight gain was associated with increased odds for macrosomia and LGA. Both lower or higher GWG increased the odds of low Apgar score.

#### Subgroup and Sensitivity Analyses

Subgroup analyses by race/ethnicity, maternal age at delivery, and infant birth year showed largely similar results with the principal results (Supplementary Table 1). Two sensitivity analyses (excluding women with cesarean section, and women with eclampsia, gestational hypertension or diabetes) confirmed the main findings (Supplementary Table 2).

## Discussion

### Principal Findings

**Figure 3:**
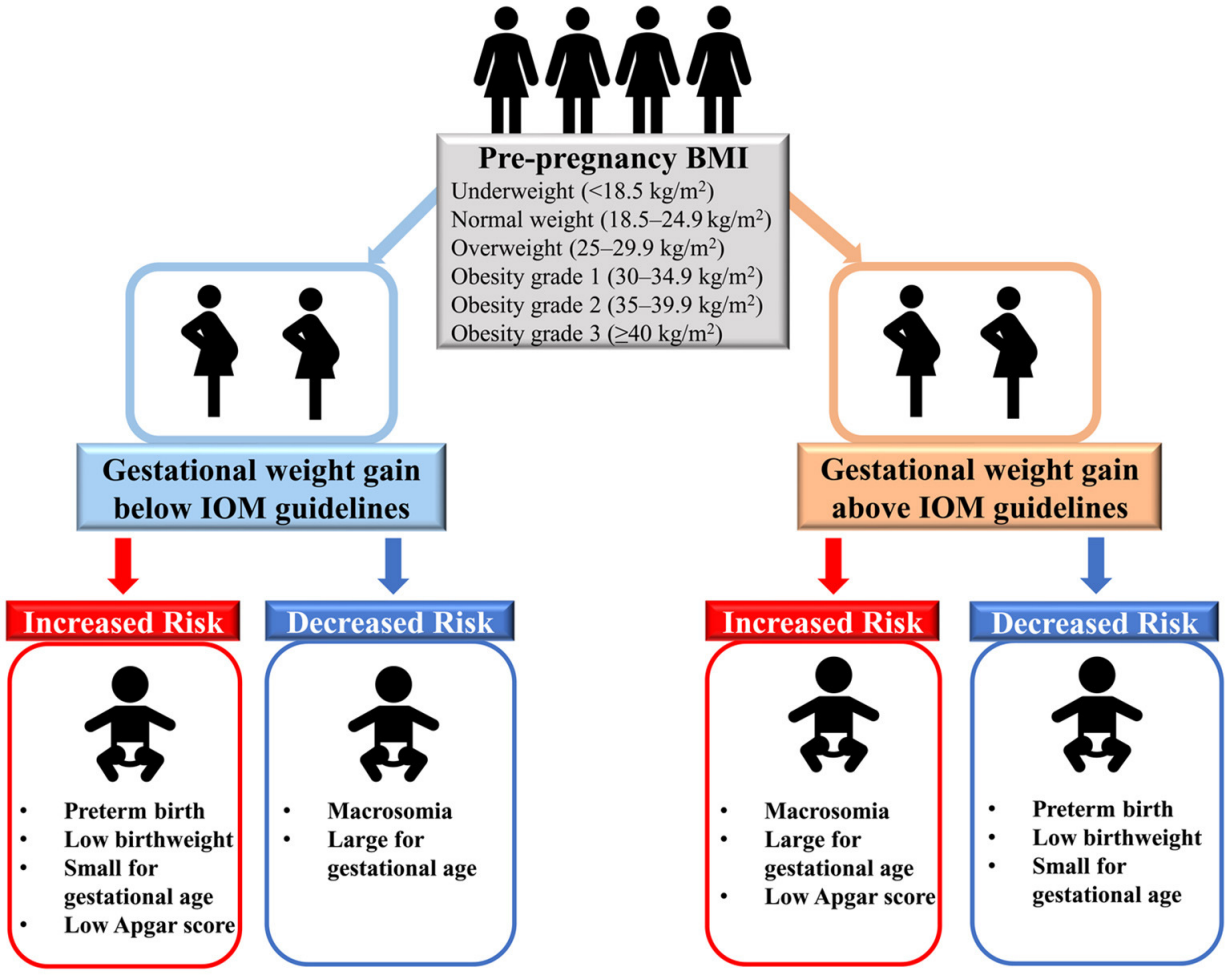
Associations between gestational weight gain and adverse birth outcomes.

In this large, multiethnic population-based retrospective cohort study of more than 9 million women with live singleton births in the U.S., we found that insufficient GWG (below IOM guidelines) was positively associated with preterm birth, low birth weight, SGA and low Apgar score, while it was inversely associated with macrosomia and LGA (Figure 3). In addition, excessive GWG (above IOM guidelines) was positively associated with macrosomia, LGA and low Apgar score, while it was inversely associated with preterm birth, low birth weight and SGA. Dose-response relationships between GWG and all six birth outcomes further confirmed the above results. Additionally, the results were similar among women with GWG below or above guidelines across pre-pregnancy BMI categories (underweight, overweight, and obesity grade 1 to grade 3). The race/ethnicity, maternal age at delivery and infant birth year had little effect on our findings.

### Compared With Previous Studies

Findings on the association between maternal GWG and adverse birth outcomes have been inconsistent. A meta-analysis of 23 studies including 1,309,136 women showed that maternal GWG below the guidelines was associated with higher odds of preterm birth (OR = 1.70, 95%CI = 1.32–2.20) and SGA (OR=1.53, 95%CI = 1.44–1.64), and lower odds of macrosomia (OR = 0.60, 95%CI = 0.52–0.68) and LGA (OR=0.59, 95%CI = 0.55–0.64); maternal GWG above the guidelines was associated with lower odds of preterm birth (OR = 0.77, 95%CI = 0.69–0.86) and SGA (OR = 0.66, 95%CI = 0.63–0.69), and higher odds of macrosomia (OR = 1.95, 95%CI = 1.79–2.11) and LGA (OR = 1.85, 95%CI = 1.76–1.95)^4^. Although parts of the results (5 outcomes) from this meta-analysis were largely similar with ours, there were several important limitations. First, there was substantial inconsistency (heterogeneity statistic *I*^2^ was from moderate to high degree) across studies included in the meta-analysis indicating a wide range of effect estimates and poor overlap in confidence intervals. Second, the definitions of infant birth outcomes (e.g., preterm birth and macrosomia) were largely different across studies that limited the comparability of results and might have, in part, explained the high *I*^2^. Third, the categories used to define GWG were different across studies which also limited comparability. Another pooled analysis of 25 cohort studies including 196,670 participants mainly from Europe also showed largely similar results with ours^2^, but it also had several limitations. First, there was significant between-study heterogeneity due to different population sources and sample sizes. Second, the subgroup analyses might have been insufficiently powered (especially for obesity grade 2 [*n* = 3,284] and obesity grade 3 [*n* = 969]). Third, confounding might exist as only three covariates (gestational age, maternal age, and parity) were considered. Fourth, they included a proportion of women who had multiple singleton pregnancies that contributed more than one data point, which may influence the generalizability of the findings. To draw a more convincing and generalizable conclusion, our study included the largest sample size of the U.S. population to date on this topic, using unified definitions of maternal GWG categories and infant birth outcomes, and adjusting for a range of potential confounding factors.

To our knowledge, few studies have assessed the associations between maternal GWG and low Apgar score, with contradictory results (17–20). A population-based retrospective cohort study of 101,259 U.S. women with chronic hypertension showed that women with GWG ≥20 lbs (i.e., 9072.0 g) above IOM guidelines were at increased odds of 5-min Apgar score <7 (OR = 1.29, 95% CI = 1.13–1.47), whereas those with GWG of 1–19 lbs (i.e., 453.6–8618.4 g) above guidelines or below guidelines were not at higher odds of low Apgar score (1–19 lbs above guideline: OR = 1.04, 95% CI = 0.93–1.17; below guideline: OR = 0.97, 95% CI = 0.84–1.11) (17). Another multi-racial population-based study of 181,948 women in Washington State suggested that maternal GWG (below or above guidelines) was not associated with Apgar score <7 at 5 min for those with ethnicity/race of white, black, east Asian, Hispanic, south Asian or other (all *P* > 0.05), except for Native American (below guidelines: OR = 3.06, 95%CI = 1.06–8.85) (18). In addition, two other studies with relatively small sample sizes (*n* = 1,709 and 1,000, respectively) did not report any association between GWG below or above guidelines and low Apgar score (19, 20). With the largest sample size on this topic to date, our study supported higher odds of low Apgar score for those with GWG above or below the guidelines.

A cohort study, including singleton live-born infants without congenital anomalies born to mothers with obesity grades 1–3, showed that weight loss was associated with an elevated risk of SGA and preterm births, and high weight gain was associated with an increased risk of LGA and preterm births (21). Consistent with our findings, similar patterns were found across obesity grades 1–3. The underlying mechanisms between GWG and adverse birth outcomes are unclear, although some evidence is available. First, insufficient GWG indicates maternal and fetal malnutrition, which may further result in adverse birth outcomes (22–24). Nevertheless, excess GWG causes the inflammatory response, high placental lipids, stress responses, and high levels of circulating estrogen, which could be exacerbated by maternal overweight and obesity (25–32). Second, insufficient or excess GWG can lead to abnormal metabolic indicators which are linked to eclampsia, gestational hypertension and gestational diabetes that can lead to adverse birth outcomes (33). Third, abnormal maternal GWG-induced gut microbiota disorders may also affect birth outcomes.

## Strengths and Limitations

Our study has several strengths. First, we used the nationwide birth certificate data with a large sample size of more than 9 million mothers from the U.S. Second, we examined a wide range of adverse birth outcomes including preterm birth, low birthweight, macrosomia, SGA, LGA, and low Apgar score. Third, we did several sensitivity analyses to confirm the robustness of the results. However, our study also has limitations. First, maternal GWG and pre-pregnancy BMI were calculated based on self-reported data, but the accuracy of self-reported information in U.S. women of reproductive age has been validated (34). Second, although we adjusted for many potential confounders, we cannot rule out residual confounding or confounding from other covariates (such as season of birth, dietary intake during pregnancy and sleep duration during pregnancy) that were not measured.

## Conclusion

Maternal GWG below or above the IOM guidelines is associated with adverse birth outcomes and the strength of the association slightly differs across maternal pre-pregnancy BMI categories. Our findings emphasize that it is important to monitor and guarantee maternal GWG within the standard range for both physicians and women themselves, and physicians should provide more tailored nutrition and exercise advice as well as prenatal care to pregnant women according to the IOM guidelines for women in different pre-pregnancy BMI categories.

## Data Availability Statement

The raw data supporting the conclusions of this article will be made available by the authors, without undue reservation.

## Ethics Statement

Ethical review and approval was not required for the study on human participants in accordance with the local legislation and institutional requirements. Written informed consent to participate in this study was provided by the participants' legal guardian/next of kin.

## Author Contributions

BX conceptualized the study. HW and LY analyzed the data, XL drafted the first draft of manuscript. All authors critically revised the manuscript for key intellectual content and approved the final version of the manuscript.

## Funding

This work was supported by the Youth Team of Humanistic and Social Science of Shandong University. The sponsor had no role in the study design, survey process, data analysis, or manuscript preparation.

## Conflict of Interest

The authors declare that the research was conducted in the absence of any commercial or financial relationships that could be construed as a potential conflict of interest.

## Publisher's Note

All claims expressed in this article are solely those of the authors and do not necessarily represent those of their affiliated organizations, or those of the publisher, the editors and the reviewers. Any product that may be evaluated in this article, or claim that may be made by its manufacturer, is not guaranteed or endorsed by the publisher.
