# Supplementary material for: Associations Between Gestational Weight Gain and Adverse Birth Outcomes: A Population-Based Retrospective Cohort Study of 9 Million Mother-Infant Pairs
Source: Front Nutr. 2022 Feb 14;9:811217. doi: 10.3389/fnut.2022.811217 (PMC8882729; doi:10.3389/fnut.2022.811217)
Supplement: Supplementary file 1 [file Table_1.DOC]

| **Supplemental Table 1. Associations of gestational weight gain below or above guidelines with infant birth outcomes by race/ethnicity, maternal age and infant birth year** | | | | | |
| --- | --- | --- | --- | --- | --- |
|  | | | | | |
|  | Total | Underweight | Normal weight | Overweight | Obesity |
| ***Hispanic*** |  |  |  |  |  |
| **Preterm birth** |  |  |  |  |  |
| Below guidelines | 1.45(1.43-1.47) | 2.14(1.99-2.30) | 1.66(1.63-1.70) | 1.33(1.30-1.37) | 1.20(1.17-1.24) |
| Within guidelines | 1.00 | 1.00 | 1.00 | 1.00 | 1.00 |
| Above guidelines | 0.85(0.84-0.86) | 0.71(0.64-0.78) | 0.76(0.74-0.78) | 0.73(0.71-0.75) | 0.84(0.82-0.86) |
| **Low birthweight** |  |  |  |  |  |
| Below guidelines | 1.40(1.37-1.42) | 1.86(1.71-2.02) | 1.48(1.43-1.52) | 1.31(1.26-1.36) | 1.18(1.13-1.23) |
| Within guidelines | 1.00 | 1.00 | 1.00 | 1.00 | 1.00 |
| Above guidelines | 0.74(0.73-0.76) | 0.66(0.58-0.74) | 0.75(0.73-0.78) | 0.77(0.74-0.79) | 0.80(0.77-0.84) |
| **Macrosomia** |  |  |  |  |  |
| Below guidelines | 0.69(0.68-0.70) | 0.47(0.38-0.58) | 0.57(0.55-0.59) | 0.68(0.66-0.71) | 0.77(0.75-0.80) |
| Within guidelines | 1.00 | 1.00 | 1.00 | 1.00 | 1.00 |
| Above guidelines | 2.02(1.99-2.05) | 2.61(2.28-2.98) | 1.95(1.90-1.99) | 1.90(1.86-1.95) | 1.63(1.59-1.67) |
| **Small for gestational age** |  |  |  |  |  |
| Below guidelines | 1.41(1.39-1.43) | 1.70(1.60-1.82) | 1.51(1.48-1.54) | 1.32(1.27-1.36) | 1.21(1.16-1.25) |
| Within guidelines | 1.00 | 1.00 | 1.00 | 1.00 | 1.00 |
| Above guidelines | 0.70(0.69-0.71) | 0.64(0.59-0.70) | 0.70(0.68-0.72) | 0.70(0.68-0.72) | 0.80(0.77-0.83) |
| **Large for gestational age** |  |  |  |  |  |
| Below guidelines | 0.76(0.75-0.78) | 0.77(0.65-0.91) | 0.66(0.64-0.68) | 0.76(0.73-0.79) | 0.80(0.78-0.83) |
| Within guidelines | 1.00 | 1.00 | 1.00 | 1.00 | 1.00 |
| Above guidelines | 1.97(1.94-2.00) | 2.45(2.12-2.83) | 1.86(1.81-1.91) | 1.86(1.82-1.91) | 1.57(1.53-1.61) |
| **Low Apgar score** |  |  |  |  |  |
| Below guidelines | 1.04(1.01-1.08) | 0.99(0.82-1.19) | 1.01(0.96-1.07) | 1.10(1.03-1.18) | 1.07(0.99-1.14) |
| Within guidelines | 1.00 | 1.00 | 1.00 | 1.00 | 1.00 |
| Above guidelines | 1.18(1.15-1.22) | 1.20(0.97-1.48) | 1.14(1.08-1.21) | 1.15(1.08-1.22) | 1.19(1.12-1.27) |
|  |  |  |  |  |  |
| ***Non-Hispanic white*** |  |  |  |  |  |
| **Preterm birth** |  |  |  |  |  |
| Below guidelines | 1.59(1.57-1.60) | 2.16(2.07-2.24) | 1.81(1.79-1.83) | 1.34(1.31-1.37) | 1.13(1.11-1.16) |
| Within guidelines | 1.00 | 1.00 | 1.00 | 1.00 | 1.00 |
| Above guidelines | 0.82(0.81-0.83) | 0.67(0.64-0.70) | 0.72(0.71-0.73) | 0.68(0.67-0.70) | 0.81(0.80-0.83) |
| **Low birthweight** |  |  |  |  |  |
| Below guidelines | 1.53(1.51-1.55) | 2.00(1.91-2.10) | 1.69(1.66-1.72) | 1.37(1.33-1.42) | 1.17(1.13-1.21) |
| Within guidelines | 1.00 | 1.00 | 1.00 | 1.00 | 1.00 |
| Above guidelines | 0.67(0.66-0.68) | 0.61(0.58-0.66) | 0.67(0.66-0.68) | 0.67(0.65-0.69) | 0.76(0.74-0.79) |
| **Macrosomia** |  |  |  |  |  |
| Below guidelines | 0.78(0.77-0.79) | 0.48(0.44-0.53) | 0.63(0.62-0.64) | 0.75(0.73-0.77) | 0.82(0.81-0.84) |
| Within guidelines | 1.00 | 1.00 | 1.00 | 1.00 | 1.00 |
| Above guidelines | 2.13(2.11-2.15) | 2.43(2.29-2.57) | 2.06(2.04-2.09) | 1.93(1.90-1.97) | 1.58(1.55-1.60) |
| **Small for gestational age** |  |  |  |  |  |
| Below guidelines | 1.52(1.50-1.53) | 1.87(1.80-1.94) | 1.66(1.64-1.69) | 1.39(1.35-1.42) | 1.20(1.17-1.24) |
| Within guidelines | 1.00 | 1.00 | 1.00 | 1.00 | 1.00 |
| Above guidelines | 0.63(0.62-0.64) | 0.60(0.57-0.63) | 0.62(0.61-0.63) | 0.62(0.61-0.63) | 0.73(0.71-0.74) |
| **Large for gestational age** |  |  |  |  |  |
| Below guidelines | 0.84(0.83-0.85) | 0.68(0.62-0.74) | 0.69(0.67-0.70) | 0.79(0.77-0.81) | 0.85(0.83-0.86) |
| Within guidelines | 1.00 | 1.00 | 1.00 | 1.00 | 1.00 |
| Above guidelines | 2.16(2.14-2.18) | 2.36(2.21-2.52) | 2.12(2.09-2.14) | 1.91(1.87-1.94) | 1.56(1.54-1.59) |
| **Low Apgar score** |  |  |  |  |  |
| Below guidelines | 1.04(1.02-1.06) | 0.93(0.84-1.02) | 1.01(0.98-1.04) | 1.05(1.00-1.10) | 1.04(1.00-1.08) |
| Within guidelines | 1.00 | 1.00 | 1.00 | 1.00 | 1.00 |
| Above guidelines | 1.16(1.14-1.18) | 1.18(1.06-1.31) | 1.12(1.09-1.15) | 1.06(1.02-1.09) | 1.05(1.02-1.09) |
|  |  |  |  |  |  |
| ***Non-Hispanic black*** |  |  |  |  |  |
| **Preterm birth** |  |  |  |  |  |
| Below guidelines | 1.47(1.45-1.49) | 2.15(2.02-2.30) | 1.74(1.70-1.78) | 1.34(1.30-1.39) | 1.15(1.12-1.19) |
| Within guidelines | 1.00 | 1.00 | 1.00 | 1.00 | 1.00 |
| Above guidelines | 0.81(0.80-0.83) | 0.66(0.60-0.72) | 0.72(0.70-0.74) | 0.73(0.71-0.75) | 0.85(0.83-0.87) |
| **Low birthweight** |  |  |  |  |  |
| Below guidelines | 1.38(1.36-1.41) | 1.73(1.60-1.86) | 1.50(1.46-1.54) | 1.33(1.27-1.38) | 1.20(1.15-1.25) |
| Within guidelines | 1.00 | 1.00 | 1.00 | 1.00 | 1.00 |
| Above guidelines | 0.71(0.70-0.73) | 0.63(0.57-0.70) | 0.69(0.67-0.71) | 0.76(0.73-0.79) | 0.81(0.78-0.85) |
| **Macrosomia** |  |  |  |  |  |
| Below guidelines | 0.77(0.75-0.79) | 0.62(0.46-0.82) | 0.65(0.61-0.68) | 0.69(0.65-0.73) | 0.79(0.76-0.83) |
| Within guidelines | 1.00 | 1.00 | 1.00 | 1.00 | 1.00 |
| Above guidelines | 2.01(1.96-2.06) | 3.06(2.51-3.74) | 2.06(1.97-2.15) | 1.91(1.83-1.99) | 1.64(1.58-1.70) |
| **Small for gestational age** |  |  |  |  |  |
| Below guidelines | 1.30(1.28-1.32) | 1.56(1.47-1.66) | 1.42(1.39-1.46) | 1.26(1.22-1.30) | 1.14(1.10-1.18) |
| Within guidelines | 1.00 | 1.00 | 1.00 | 1.00 | 1.00 |
| Above guidelines | 0.69(0.68-0.70) | 0.60(0.55-0.65) | 0.67(0.65-0.69) | 0.71(0.69-0.73) | 0.78(0.76-0.81) |
| **Large for gestational age** |  |  |  |  |  |
| Below guidelines | 0.85(0.83-0.88) | 1.02(0.81-1.27) | 0.86(0.81-0.90) | 0.76(0.72-0.81) | 0.80(0.77-0.84) |
| Within guidelines | 1.00 | 1.00 | 1.00 | 1.00 | 1.00 |
| Above guidelines | 1.91(1.86-1.95) | 2.45(2.01-2.98) | 1.99(1.90-2.08) | 1.74(1.67-1.82) | 1.55(1.50-1.60) |
| **Low Apgar score** |  |  |  |  |  |
| Below guidelines | 1.04(1.01-1.07) | 1.03(0.88-1.21) | 1.02(0.97-1.07) | 1.06(1.00-1.13) | 1.04(0.98-1.09) |
| Within guidelines | 1.00 | 1.00 | 1.00 | 1.00 | 1.00 |
| Above guidelines | 1.11(1.08-1.15) | 1.25(1.04-1.50) | 1.12(1.06-1.18) | 1.06(1.00-1.12) | 1.06(1.00-1.11) |
|  |  |  |  |  |  |
| ***Other*** |  |  |  |  |  |
| **Preterm birth** |  |  |  |  |  |
| Below guidelines | 1.50(1.47-1.53) | 1.98(1.83-2.14) | 1.69(1.65-1.74) | 1.26(1.20-1.32) | 1.10(1.04-1.16) |
| Within guidelines | 1.00 | 1.00 | 1.00 | 1.00 | 1.00 |
| Above guidelines | 0.83(0.81-0.84) | 0.76(0.67-0.85) | 0.70(0.68-0.73) | 0.70(0.67-0.73) | 0.81(0.77-0.85) |
| **Low birthweight** |  |  |  |  |  |
| Below guidelines | 1.47(1.43-1.51) | 1.74(1.60-1.89) | 1.58(1.53-1.64) | 1.27(1.19-1.35) | 1.12(1.03-1.22) |
| Within guidelines | 1.00 | 1.00 | 1.00 | 1.00 | 1.00 |
| Above guidelines | 0.69(0.67-0.71) | 0.67(0.59-0.76) | 0.72(0.69-0.75) | 0.67(0.64-0.71) | 0.74(0.68-0.79) |
| **Macrosomia** |  |  |  |  |  |
| Below guidelines | 0.68(0.66-0.70) | 0.40(0.32-0.49) | 0.57(0.55-0.60) | 0.64(0.60-0.69) | 0.85(0.80-0.90) |
| Within guidelines | 1.00 | 1.00 | 1.00 | 1.00 | 1.00 |
| Above guidelines | 2.44(2.38-2.49) | 2.65(2.29-3.07) | 2.27(2.19-2.35) | 2.09(2.00-2.19) | 1.79(1.70-1.88) |
| **Small for gestational age** |  |  |  |  |  |
| Below guidelines | 1.48(1.45-1.51) | 1.81(1.70-1.92) | 1.57(1.53-1.61) | 1.29(1.23-1.35) | 1.13(1.06-1.21) |
| Within guidelines | 1.00 | 1.00 | 1.00 | 1.00 | 1.00 |
| Above guidelines | 0.66(0.65-0.68) | 0.63(0.58-0.69) | 0.67(0.65-0.69) | 0.66(0.63-0.69) | 0.73(0.68-0.78) |
| **Large for gestational age** |  |  |  |  |  |
| Below guidelines | 0.75(0.72-0.77) | 0.49(0.40-0.61) | 0.65(0.62-0.68) | 0.74(0.69-0.79) | 0.85(0.80-0.91) |
| Within guidelines | 1.00 | 1.00 | 1.00 | 1.00 | 1.00 |
| Above guidelines | 2.42(2.36-2.48) | 2.58(2.19-3.05) | 2.26(2.18-2.35) | 2.00(1.91-2.10) | 1.74(1.66-1.83) |
| **Low Apgar score** |  |  |  |  |  |
| Below guidelines | 1.05(1.00-1.10) | 1.19(0.97-1.45) | 1.03(0.97-1.11) | 1.08(0.97-1.19) | 1.06(0.94-1.19) |
| Within guidelines | 1.00 | 1.00 | 1.00 | 1.00 | 1.00 |
| Above guidelines | 1.29(1.24-1.36) | 1.38(1.09-1.75) | 1.26(1.18-1.35) | 1.10(1.00-1.20) | 1.19(1.08-1.33) |
|  |  |  |  |  |  |
| ***18-29 years*** |  |  |  |  |  |
| **Preterm birth** |  |  |  |  |  |
| Below guidelines | 1.57(1.56-1.58) | 2.18(2.11-2.26) | 1.79(1.77-1.81) | 1.37(1.34-1.40) | 1.18(1.16-1.20) |
| Within guidelines | 1.00 | 1.00 | 1.00 | 1.00 | 1.00 |
| Above guidelines | 0.79(0.79-0.80) | 0.66(0.63-0.69) | 0.70(0.69-0.71) | 0.70(0.69-0.71) | 0.82(0.80-0.83) |
| **Low birthweight** |  |  |  |  |  |
| Below guidelines | 1.46(1.45-1.48) | 1.88(1.80-1.95) | 1.59(1.56-1.61) | 1.33(1.30-1.37) | 1.15(1.12-1.19) |
| Within guidelines | 1.00 | 1.00 | 1.00 | 1.00 | 1.00 |
| Above guidelines | 0.68(0.67-0.69) | 0.61(0.58-0.65) | 0.69(0.67-0.70) | 0.71(0.69-0.73) | 0.78(0.76-0.80) |
| **Macrosomia** |  |  |  |  |  |
| Below guidelines | 0.77(0.76-0.78) | 0.48(0.43-0.53) | 0.61(0.60-0.63) | 0.73(0.71-0.75) | 0.82(0.80-0.83) |
| Within guidelines | 1.00 | 1.00 | 1.00 | 1.00 | 1.00 |
| Above guidelines | 2.17(2.15-2.19) | 2.65(2.48-2.82) | 2.12(2.09-2.15) | 2.01(1.97-2.04) | 1.62(1.60-1.65) |
| **Small for gestational age** |  |  |  |  |  |
| Below guidelines | 1.42(1.41-1.43) | 1.73(1.68-1.78) | 1.55(1.53-1.57) | 1.31(1.28-1.33) | 1.18(1.15-1.20) |
| Within guidelines | 1.00 | 1.00 | 1.00 | 1.00 | 1.00 |
| Above guidelines | 0.65(0.65-0.66) | 0.59(0.57-0.61) | 0.65(0.64-0.66) | 0.65(0.64-0.67) | 0.76(0.75-0.78) |
| **Large for gestational age** |  |  |  |  |  |
| Below guidelines | 0.84(0.83-0.85) | 0.76(0.69-0.83) | 0.72(0.70-0.73) | 0.79(0.77-0.81) | 0.84(0.82-0.86) |
| Within guidelines | 1.00 | 1.00 | 1.00 | 1.00 | 1.00 |
| Above guidelines | 2.15(2.13-2.17) | 2.45(2.28-2.62) | 2.12(2.08-2.15) | 1.94(1.90-1.98) | 1.59(1.56-1.61) |
| **Low Apgar score** |  |  |  |  |  |
| Below guidelines | 1.04(1.02-1.06) | 1.03(0.95-1.12) | 1.01(0.99-1.04) | 1.07(1.03-1.12) | 1.04(1.00-1.08) |
| Within guidelines | 1.00 | 1.00 | 1.00 | 1.00 | 1.00 |
| Above guidelines | 1.17(1.15-1.19) | 1.31(1.20-1.44) | 1.14(1.11-1.17) | 1.09(1.05-1.13) | 1.09(1.05-1.12) |
|  |  |  |  |  |  |
| ***30-39 years*** |  |  |  |  |  |
| **Preterm birth** |  |  |  |  |  |
| Below guidelines | 1.46(1.45-1.48) | 2.00(1.89-2.11) | 1.72(1.69-1.74) | 1.28(1.25-1.30) | 1.12(1.10-1.14) |
| Within guidelines | 1.00 | 1.00 | 1.00 | 1.00 | 1.00 |
| Above guidelines | 0.86(0.85-0.87) | 0.76(0.71-0.82) | 0.75(0.74-0.76) | 0.70(0.69-0.72) | 0.83(0.81-0.84) |
| **Low birthweight** |  |  |  |  |  |
| Below guidelines | 1.46(1.44-1.48) | 1.90(1.78-2.03) | 1.61(1.57-1.64) | 1.34(1.29-1.38) | 1.21(1.17-1.26) |
| Within guidelines | 1.00 | 1.00 | 1.00 | 1.00 | 1.00 |
| Above guidelines | 0.71(0.70-0.72) | 0.68(0.62-0.75) | 0.70(0.69-0.72) | 0.71(0.69-0.73) | 0.79(0.77-0.82) |
| **Macrosomia** |  |  |  |  |  |
| Below guidelines | 0.74(0.73-0.75) | 0.48(0.43-0.53) | 0.61(0.60-0.62) | 0.71(0.69-0.73) | 0.80(0.79-0.82) |
| Within guidelines | 1.00 | 1.00 | 1.00 | 1.00 | 1.00 |
| Above guidelines | 2.08(2.06-2.09) | 2.31(2.13-2.50) | 2.01(1.98-2.04) | 1.87(1.84-1.91) | 1.58(1.55-1.61) |
| **Small for gestational age** |  |  |  |  |  |
| Below guidelines | 1.46(1.45-1.48) | 1.88(1.79-1.97) | 1.60(1.58-1.63) | 1.34(1.31-1.38) | 1.19(1.15-1.22) |
| Within guidelines | 1.00 | 1.00 | 1.00 | 1.00 | 1.00 |
| Above guidelines | 0.68(0.67-0.68) | 0.68(0.63-0.72) | 0.66(0.65-0.67) | 0.67(0.66-0.69) | 0.76(0.74-0.78) |
| **Large for gestational age** |  |  |  |  |  |
| Below guidelines | 0.80(0.79-0.81) | 0.63(0.57-0.70) | 0.67(0.66-0.68) | 0.77(0.74-0.79) | 0.82(0.81-0.84) |
| Within guidelines | 1.00 | 1.00 | 1.00 | 1.00 | 1.00 |
| Above guidelines | 2.10(2.08-2.12) | 2.35(2.15-2.57) | 2.06(2.03-2.09) | 1.85(1.82-1.89) | 1.55(1.53-1.58) |
| **Low Apgar score** |  |  |  |  |  |
| Below guidelines | 1.04(1.01-1.06) | 0.87(0.76-1.00) | 1.01(0.97-1.04) | 1.06(1.01-1.12) | 1.06(1.01-1.11) |
| Within guidelines | 1.00 | 1.00 | 1.00 | 1.00 | 1.00 |
| Above guidelines | 1.16(1.13-1.18) | 0.99(0.84-1.17) | 1.13(1.09-1.17) | 1.06(1.02-1.11) | 1.08(1.04-1.13) |
|  |  |  |  |  |  |
| ***40-49 years*** |  |  |  |  |  |
| **Preterm birth** |  |  |  |  |  |
| Below guidelines | 1.39(1.34-1.44) | 1.76(1.41-2.19) | 1.60(1.52-1.68) | 1.28(1.20-1.37) | 1.15(1.08-1.23) |
| Within guidelines | 1.00 | 1.00 | 1.00 | 1.00 | 1.00 |
| Above guidelines | 0.95(0.92-0.98) | 0.57(0.41-0.81) | 0.85(0.80-0.90) | 0.79(0.74-0.83) | 0.94(0.89-1.00) |
| **Low birthweight** |  |  |  |  |  |
| Below guidelines | 1.36(1.30-1.43) | 1.84(1.40-2.41) | 1.49(1.39-1.60) | 1.21(1.10-1.33) | 1.18(1.07-1.31) |
| Within guidelines | 1.00 | 1.00 | 1.00 | 1.00 | 1.00 |
| Above guidelines | 0.68(0.65-0.72) | 0.50(0.33-0.77) | 0.65(0.59-0.71) | 0.71(0.65-0.77) | 0.77(0.7-0.85) |
| **Macrosomia** |  |  |  |  |  |
| Below guidelines | 0.72(0.68-0.75) | 0.34(0.19-0.58) | 0.63(0.58-0.67) | 0.63(0.57-0.70) | 0.83(0.77-0.90) |
| Within guidelines | 1.00 | 1.00 | 1.00 | 1.00 | 1.00 |
| Above guidelines | 2.05(1.98-2.12) | 2.43(1.67-3.55) | 1.90(1.80-2.01) | 1.84(1.73-1.96) | 1.76(1.65-1.88) |
| **Small for gestational age** |  |  |  |  |  |
| Below guidelines | 1.40(1.34-1.46) | 1.77(1.44-2.16) | 1.56(1.47-1.65) | 1.30(1.19-1.41) | 1.13(1.02-1.24) |
| Within guidelines | 1.00 | 1.00 | 1.00 | 1.00 | 1.00 |
| Above guidelines | 0.69(0.66-0.72) | 0.62(0.46-0.85) | 0.66(0.62-0.71) | 0.69(0.64-0.74) | 0.74(0.68-0.81) |
| **Large for gestational age** |  |  |  |  |  |
| Below guidelines | 0.75(0.72-0.79) | 0.47(0.29-0.77) | 0.66(0.61-0.71) | 0.69(0.63-0.76) | 0.82(0.77-0.89) |
| Within guidelines | 1.00 | 1.00 | 1.00 | 1.00 | 1.00 |
| Above guidelines | 1.98(1.92-2.05) | 2.05(1.37-3.05) | 1.85(1.75-1.96) | 1.78(1.67-1.89) | 1.65(1.56-1.75) |
| **Low Apgar score** |  |  |  |  |  |
| Below guidelines | 1.09(1.01-1.18) | 0.99(0.57-1.70) | 1.04(0.92-1.18) | 1.11(0.95-1.30) | 1.10(0.96-1.27) |
| Within guidelines | 1.00 | 1.00 | 1.00 | 1.00 | 1.00 |
| Above guidelines | 1.18(1.10-1.27) | 0.93(0.47-1.87) | 1.10(0.96-1.25) | 1.16(1.02-1.32) | 1.08(0.95-1.23) |
|  |  |  |  |  |  |
| ***2016*** |  |  |  |  |  |
| **Preterm birth** |  |  |  |  |  |
| Below guidelines | 1.53(1.51-1.55) | 2.20(2.10-2.30) | 1.76(1.73-1.78) | 1.33(1.30-1.36) | 1.15(1.13-1.18) |
| Within guidelines | 1.00 | 1.00 | 1.00 | 1.00 | 1.00 |
| Above guidelines | 0.82(0.81-0.83) | 0.66(0.62-0.70) | 0.72(0.70-0.73) | 0.69(0.68-0.71) | 0.83(0.82-0.85) |
| **Low birthweight** |  |  |  |  |  |
| Below guidelines | 1.46(1.44-1.48) | 1.88(1.78-1.98) | 1.60(1.57-1.64) | 1.31(1.26-1.35) | 1.15(1.10-1.19) |
| Within guidelines | 1.00 | 1.00 | 1.00 | 1.00 | 1.00 |
| Above guidelines | 0.70(0.69-0.71) | 0.65(0.60-0.70) | 0.69(0.68-0.71) | 0.71(0.69-0.74) | 0.79(0.76-0.82) |
| **Macrosomia** |  |  |  |  |  |
| Below guidelines | 0.75(0.74-0.76) | 0.50(0.45-0.57) | 0.62(0.60-0.63) | 0.72(0.70-0.74) | 0.82(0.80-0.84) |
| Within guidelines | 1.00 | 1.00 | 1.00 | 1.00 | 1.00 |
| Above guidelines | 2.13(2.11-2.15) | 2.57(2.37-2.78) | 2.05(2.02-2.08) | 1.93(1.89-1.97) | 1.62(1.59-1.65) |
| **Small for gestational age** |  |  |  |  |  |
| Below guidelines | 1.44(1.43-1.46) | 1.82(1.74-1.90) | 1.57(1.54-1.59) | 1.30(1.26-1.33) | 1.19(1.15-1.22) |
| Within guidelines | 1.00 | 1.00 | 1.00 | 1.00 | 1.00 |
| Above guidelines | 0.67(0.66-0.67) | 0.64(0.61-0.68) | 0.65(0.64-0.66) | 0.66(0.64-0.68) | 0.76(0.74-0.78) |
| **Large for gestational age** |  |  |  |  |  |
| Below guidelines | 0.82(0.80-0.83) | 0.71(0.64-0.80) | 0.69(0.67-0.71) | 0.77(0.75-0.80) | 0.84(0.82-0.86) |
| Within guidelines | 1.00 | 1.00 | 1.00 | 1.00 | 1.00 |
| Above guidelines | 2.13(2.11-2.16) | 2.43(2.23-2.65) | 2.07(2.04-2.11) | 1.89(1.85-1.93) | 1.58(1.55-1.61) |
| **Low Apgar score** |  |  |  |  |  |
| Below guidelines | 1.02(1.00-1.05) | 1.04(0.92-1.17) | 1.01(0.97-1.05) | 1.03(0.98-1.09) | 1.02(0.97-1.08) |
| Within guidelines | 1.00 | 1.00 | 1.00 | 1.00 | 1.00 |
| Above guidelines | 1.17(1.15-1.20) | 1.29(1.13-1.47) | 1.14(1.11-1.18) | 1.09(1.04-1.14) | 1.10(1.05-1.15) |
|  |  |  |  |  |  |
| ***2017*** |  |  |  |  |  |
| **Preterm birth** |  |  |  |  |  |
| Below guidelines | 1.53(1.52-1.55) | 2.08(1.98-2.19) | 1.76(1.73-1.79) | 1.34(1.31-1.38) | 1.17(1.14-1.20) |
| Within guidelines | 1.00 | 1.00 | 1.00 | 1.00 | 1.00 |
| Above guidelines | 0.83(0.82-0.84) | 0.67(0.63-0.72) | 0.73(0.71-0.74) | 0.71(0.70-0.73) | 0.82(0.81-0.84) |
| **Low birthweight** |  |  |  |  |  |
| Below guidelines | 1.47(1.44-1.49) | 1.89(1.79-2.01) | 1.59(1.56-1.63) | 1.35(1.30-1.40) | 1.18(1.14-1.23) |
| Within guidelines | 1.00 | 1.00 | 1.00 | 1.00 | 1.00 |
| Above guidelines | 0.69(0.68-0.70) | 0.60(0.55-0.65) | 0.70(0.68-0.72) | 0.72(0.70-0.74) | 0.78(0.76-0.81) |
| **Macrosomia** |  |  |  |  |  |
| Below guidelines | 0.75(0.74-0.76) | 0.50(0.44-0.57) | 0.61(0.60-0.63) | 0.72(0.70-0.75) | 0.81(0.79-0.83) |
| Within guidelines | 1.00 | 1.00 | 1.00 | 1.00 | 1.00 |
| Above guidelines | 2.13(2.11-2.16) | 2.67(2.45-2.91) | 2.08(2.05-2.12) | 1.95(1.90-1.99) | 1.60(1.57-1.63) |
| **Small for gestational age** |  |  |  |  |  |
| Below guidelines | 1.44(1.42-1.46) | 1.75(1.68-1.84) | 1.57(1.55-1.60) | 1.34(1.30-1.38) | 1.18(1.14-1.22) |
| Within guidelines | 1.00 | 1.00 | 1.00 | 1.00 | 1.00 |
| Above guidelines | 0.66(0.65-0.67) | 0.56(0.53-0.60) | 0.65(0.64-0.67) | 0.67(0.66-0.69) | 0.76(0.74-0.78) |
| **Large for gestational age** |  |  |  |  |  |
| Below guidelines | 0.82(0.81-0.83) | 0.73(0.65-0.83) | 0.69(0.67-0.71) | 0.77(0.74-0.80) | 0.84(0.81-0.86) |
| Within guidelines | 1.00 | 1.00 | 1.00 | 1.00 | 1.00 |
| Above guidelines | 2.13(2.10-2.15) | 2.49(2.26-2.74) | 2.10(2.06-2.14) | 1.89(1.85-1.94) | 1.57(1.54-1.60) |
| **Low Apgar score** |  |  |  |  |  |
| Below guidelines | 1.06(1.03-1.09) | 0.91(0.80-1.03) | 1.03(0.99-1.07) | 1.11(1.05-1.17) | 1.06(1.01-1.12) |
| Within guidelines | 1.00 | 1.00 | 1.00 | 1.00 | 1.00 |
| Above guidelines | 1.18(1.16-1.21) | 1.15(1.01-1.32) | 1.15(1.11-1.20) | 1.12(1.07-1.17) | 1.10(1.05-1.15) |
|  |  |  |  |  |  |
| ***2018*** |  |  |  |  |  |
| **Preterm birth** |  |  |  |  |  |
| Below guidelines | 1.50(1.48-1.52) | 2.08(1.98-2.19) | 1.75(1.72-1.78) | 1.32(1.28-1.35) | 1.14(1.11-1.17) |
| Within guidelines | 1.00 | 1.00 | 1.00 | 1.00 | 1.00 |
| Above guidelines | 0.83(0.82-0.84) | 0.72(0.67-0.77) | 0.73(0.71-0.74) | 0.71(0.69-0.72) | 0.82(0.80-0.84) |
| **Low birthweight** |  |  |  |  |  |
| Below guidelines | 1.45(1.43-1.47) | 1.88(1.77-1.99) | 1.58(1.54-1.62) | 1.33(1.29-1.38) | 1.21(1.16-1.25) |
| Within guidelines | 1.00 | 1.00 | 1.00 | 1.00 | 1.00 |
| Above guidelines | 0.68(0.67-0.69) | 0.63(0.58-0.68) | 0.68(0.67-0.70) | 0.70(0.67-0.72) | 0.78(0.76-0.81) |
| **Macrosomia** |  |  |  |  |  |
| Below guidelines | 0.75(0.74-0.76) | 0.42(0.36-0.48) | 0.61(0.59-0.62) | 0.71(0.68-0.73) | 0.81(0.79-0.83) |
| Within guidelines | 1.00 | 1.00 | 1.00 | 1.00 | 1.00 |
| Above guidelines | 2.10(2.08-2.12) | 2.31(2.11-2.53) | 2.06(2.02-2.09) | 1.92(1.88-1.96) | 1.60(1.56-1.63) |
| **Small for gestational age** |  |  |  |  |  |
| Below guidelines | 1.43(1.41-1.44) | 1.73(1.65-1.81) | 1.57(1.54-1.60) | 1.33(1.29-1.37) | 1.17(1.13-1.21) |
| Within guidelines | 1.00 | 1.00 | 1.00 | 1.00 | 1.00 |
| Above guidelines | 0.66(0.65-0.67) | 0.62(0.58-0.66) | 0.65(0.64-0.66) | 0.65(0.63-0.67) | 0.76(0.74-0.78) |
| **Large for gestational age** |  |  |  |  |  |
| Below guidelines | 0.82(0.80-0.83) | 0.64(0.56-0.72) | 0.69(0.67-0.71) | 0.78(0.75-0.81) | 0.82(0.80-0.84) |
| Within guidelines | 1.00 | 1.00 | 1.00 | 1.00 | 1.00 |
| Above guidelines | 2.10(2.08-2.13) | 2.27(2.06-2.51) | 2.07(2.03-2.11) | 1.89(1.84-1.93) | 1.57(1.53-1.60) |
| **Low Apgar score** |  |  |  |  |  |
| Below guidelines | 1.04(1.02-1.07) | 1.00(0.88-1.14) | 0.99(0.96-1.03) | 1.07(1.01-1.13) | 1.06(1.01-1.11) |
| Within guidelines | 1.00 | 1.00 | 1.00 | 1.00 | 1.00 |
| Above guidelines | 1.14(1.12-1.17) | 1.22(1.06-1.41) | 1.12(1.08-1.16) | 1.04(1.00-1.09) | 1.07(1.02-1.12) |

Logistic regression models were adjusted for maternal age at delivery, race/ethnicity, education level, marital status, smoking status during pregnancy, parity, infant sex, and total number of prenatal care visits.

Gestational age was additionally adjusted for three outcomes including low birthweight/macrosomia/low Apgar score.

| **Supplemental Table 2. Sensitivity analyses of the associations of gestational weight gain below or above guidelines with infant birth outcomes** | | | | | |
| --- | --- | --- | --- | --- | --- |
|  | | | | | |
|  | Total | Underweight | Normal weight | Overweight | Obesity |
| ***Excluding women with caesarean section*** |  |  |  |  |  |
| **Preterm birth** |  |  |  |  |  |
| Below guidelines | 1.55(1.53-1.56) | 2.10(2.03-2.17) | 1.74(1.72-1.76) | 1.35(1.32-1.37) | 1.17(1.15-1.19) |
| Within guidelines | 1.00 | 1.00 | 1.00 | 1.00 | 1.00 |
| Above guidelines | 0.80(0.79-0.81) | 0.67(0.64-0.70) | 0.71(0.70-0.72) | 0.70(0.69-0.71) | 0.80(0.79-0.81) |
| **Low birthweight** |  |  |  |  |  |
| Below guidelines | 1.50(1.49-1.52) | 1.91(1.84-1.99) | 1.62(1.59-1.64) | 1.36(1.32-1.39) | 1.18(1.15-1.22) |
| Within guidelines | 1.00 | 1.00 | 1.00 | 1.00 | 1.00 |
| Above guidelines | 0.67(0.67-0.68) | 0.62(0.58-0.65) | 0.68(0.67-0.69) | 0.70(0.68-0.71) | 0.78(0.76-0.80) |
| **Macrosomia** |  |  |  |  |  |
| Below guidelines | 0.73(0.72-0.74) | 0.48(0.44-0.52) | 0.61(0.60-0.62) | 0.72(0.71-0.74) | 0.82(0.80-0.83) |
| Within guidelines | 1.00 | 1.00 | 1.00 | 1.00 | 1.00 |
| Above guidelines | 2.00(1.99-2.02) | 2.47(2.33-2.61) | 1.98(1.96-2.00) | 1.86(1.83-1.89) | 1.53(1.51-1.56) |
| **Small for gestational age** |  |  |  |  |  |
| Below guidelines | 1.46(1.45-1.47) | 1.76(1.71-1.81) | 1.57(1.55-1.59) | 1.34(1.31-1.36) | 1.19(1.16-1.22) |
| Within guidelines | 1.00 | 1.00 | 1.00 | 1.00 | 1.00 |
| Above guidelines | 0.65(0.65-0.66) | 0.61(0.58-0.63) | 0.65(0.64-0.66) | 0.67(0.65-0.68) | 0.76(0.74-0.78) |
| **Large for gestational age** |  |  |  |  |  |
| Below guidelines | 0.79(0.78-0.80) | 0.65(0.60-0.71) | 0.67(0.66-0.69) | 0.77(0.75-0.79) | 0.84(0.82-0.86) |
| Within guidelines | 1.00 | 1.00 | 1.00 | 1.00 | 1.00 |
| Above guidelines | 2.02(2.00-2.03) | 2.33(2.18-2.48) | 2.01(1.98-2.03) | 1.83(1.80-1.86) | 1.50(1.47-1.52) |
| **Low Apgar score** |  |  |  |  |  |
| Below guidelines | 1.05(1.03-1.07) | 0.99(0.90-1.09) | 1.01(0.98-1.04) | 1.11(1.07-1.16) | 1.07(1.03-1.12) |
| Within guidelines | 1.00 | 1.00 | 1.00 | 1.00 | 1.00 |
| Above guidelines | 1.20(1.18-1.23) | 1.26(1.14-1.39) | 1.17(1.14-1.21) | 1.13(1.09-1.17) | 1.13(1.09-1.17) |
|  |  |  |  |  |  |
| ***Excluding women with eclampsia, gestational hypertension or diabetes*** |  |  |  |  |  |
| **Preterm birth** |  |  |  |  |  |
| Below guidelines | 1.59(1.58-1.60) | 2.18(2.12-2.25) | 1.82(1.80-1.84) | 1.38(1.36-1.40) | 1.21(1.19-1.23) |
| Within guidelines | 1.00 | 1.00 | 1.00 | 1.00 | 1.00 |
| Above guidelines | 0.74(0.74-0.75) | 0.65(0.62-0.68) | 0.67(0.66-0.68) | 0.66(0.65-0.66) | 0.76(0.75-0.77) |
| **Low birthweight** |  |  |  |  |  |
| Below guidelines | 1.52(1.50-1.53) | 1.92(1.85-1.99) | 1.64(1.62-1.66) | 1.37(1.34-1.4) | 1.19(1.16-1.22) |
| Within guidelines | 1.00 | 1.00 | 1.00 | 1.00 | 1.00 |
| Above guidelines | 0.67(0.67-0.68) | 0.62(0.59-0.66) | 0.67(0.66-0.68) | 0.69(0.68-0.71) | 0.76(0.75-0.78) |
| **Macrosomia** |  |  |  |  |  |
| Below guidelines | 0.74(0.73-0.75) | 0.47(0.44-0.51) | 0.61(0.61-0.62) | 0.73(0.71-0.74) | 0.82(0.80-0.83) |
| Within guidelines | 1.00 | 1.00 | 1.00 | 1.00 | 1.00 |
| Above guidelines | 2.12(2.11-2.14) | 2.52(2.39-2.65) | 2.06(2.04-2.08) | 1.93(1.91-1.96) | 1.60(1.58-1.62) |
| **Small for gestational age** |  |  |  |  |  |
| Below guidelines | 1.46(1.45-1.47) | 1.78(1.73-1.82) | 1.58(1.56-1.60) | 1.32(1.30-1.34) | 1.19(1.16-1.21) |
| Within guidelines | 1.00 | 1.00 | 1.00 | 1.00 | 1.00 |
| Above guidelines | 0.65(0.64-0.66) | 0.60(0.58-0.63) | 0.64(0.64-0.65) | 0.65(0.64-0.66) | 0.75(0.73-0.76) |
| **Large for gestational age** |  |  |  |  |  |
| Below guidelines | 0.80(0.80-0.81) | 0.69(0.64-0.74) | 0.69(0.68-0.70) | 0.78(0.77-0.80) | 0.83(0.82-0.85) |
| Within guidelines | 1.00 | 1.00 | 1.00 | 1.00 | 1.00 |
| Above guidelines | 2.13(2.12-2.15) | 2.39(2.26-2.53) | 2.09(2.06-2.11) | 1.90(1.87-1.93) | 1.57(1.55-1.59) |
| **Low Apgar score** |  |  |  |  |  |
| Below guidelines | 1.04(1.03-1.06) | 0.97(0.90-1.05) | 1.02(0.99-1.04) | 1.08(1.04-1.12) | 1.05(1.01-1.08) |
| Within guidelines | 1.00 | 1.00 | 1.00 | 1.00 | 1.00 |
| Above guidelines | 1.16(1.15-1.18) | 1.20(1.11-1.31) | 1.14(1.12-1.17) | 1.08(1.05-1.11) | 1.07(1.04-1.11) |

Logistic regression models were adjusted for maternal age at delivery, race/ethnicity, education level, marital status, smoking status during pregnancy, parity, infant sex, and total number of prenatal care visits.

Gestational age was additionally adjusted for three outcomes including low birthweight/macrosomia/low Apgar score.
